# High-Dose-Rate Brachytherapy Combined with External Beam Radiotherapy for Newly Defined Very High-Risk and Regional Prostate Cancer: A 17-Year Single-Institution Experience

**DOI:** 10.3390/cancers18040595

**Published:** 2026-02-11

**Authors:** Tomoyuki Makino, Takayuki Sakurai, Shigeyuki Takamatsu, Ryunosuke Nakagawa, Taiki Kamijima, Hiroshi Kano, Renato Naito, Hiroaki Iwamoto, Hiroshi Yaegashi, Kazuyoshi Shigehara, Takahiro Nohara, Kouji Izumi, Atsushi Mizokami

**Affiliations:** 1 Department of Integrative Cancer Therapy and Urology, Kanazawa University Graduate School of Medical Science, 13-1 Takara-Machi, Kanazawa 920-8641, Japan; r_a_rhero0226southern@yahoo.co.jp (R.N.); kamiji0029@yahoo.co.jp (T.K.); kanazawa_iimati@yahoo.co.jp (H.K.); thealfuu@yahoo.co.jp (R.N.); hiroaki017@yahoo.co.jp (H.I.); hyae2002jp@yahoo.co.jp (H.Y.); kshigehara0415@yahoo.co.jp (K.S.); t_nohara704@yahoo.co.jp (T.N.); azuizu2003@yahoo.co.jp (K.I.); mizokami@staff.kanazawa-u.ac.jp (A.M.); 2 Department of Radiology, Kanazawa University Graduate School of Medical Science, 13-1 Takara-Machi, Kanazawa 920-8641, Japan; tkyk81@gmail.com (T.S.); shigerad@staff.kanazawa-u.ac.jp (S.T.)

**Keywords:** high-dose-rate brachytherapy, prostate cancer, very high-risk, locally advanced, regional disease, NCCN Guidelines, radiotherapy, androgen deprivation therapy, prognosis

## Abstract

**Background**: The updated National Comprehensive Cancer Network (NCCN) criteria redefine the very high-risk (VHR) prostate cancer (PC) category, identifying patients with highly aggressive disease. Evidence regarding the outcomes of high-dose-rate brachytherapy (HDR-BT) in these patients, including those with regional disease, is limited. Therefore, the present study focused on analyzing the long-term clinical performance of HDR-BT-based radiotherapy (RT) for patients meeting the NCCN criteria for VHR or regional PC. **Methods**: A total of 215 patients with VHR (*n* = 179) or regional disease (*n* = 36) treated with HDR-BT with external beam RT between 2006 and 2022 were retrospectively reviewed. Recurrence-free survival (RFS), cancer-specific survival (CSS), and overall survival (OS) were assessed using Kaplan–Meier methods, and prognostic factors were analyzed using Cox regression. **Results**: Recurrence occurred in 19 (8.8%) patients, and 22 (10.2%) died, including 4 PC-specific deaths. The 5-year RFS, CSS, and OS rates were 92.5%, 98.9%, and 96.2%, respectively, and the 8-year rates were 88.5%, 97.8%, and 90.8%, respectively. Multivariate analysis revealed that pre-RT prostate-specific antigen (PSA) > 0.1 ng/mL (HR 3.93; *p* = 0.010) and cT4 disease (HR 4.49; *p* = 0.032) were independent predictors of inferior RFS. Grade ≥ 3 genitourinary toxicity was observed in 1.9% of patients, and no Grade ≥ 3 gastrointestinal toxicity occurred. **Conclusions**: HDR-BT-based RT provides durable disease control and low toxicity in patients with NCCN-defined VHR and regional PC. Pre-RT PSA and T stage may inform risk-adapted treatment strategies. The present findings demonstrate the importance of HDR-BT as an effective component of multimodal therapy for highly aggressive PC.

## Simple Summary

This study investigated the effectiveness of high-dose-rate brachytherapy (HDR-BT) combined with external radiation in controlling very aggressive forms of prostate cancer (PC), including cases with tumor spread to nearby lymph nodes. We analyzed the long-term clinical results of an over 200-patient cohort treated with combined HDR-BT and external beam radiotherapy. This study specifically utilizes the revised NCCN criteria to focus on those classified within the highest-risk categories at our institution. This approach provided strong cancer control for many years, with low recurrence rates and very few serious adverse events. Patients with cancer that had grown outside the prostate or with insufficient pre-HDR-BT prostate-specific antigen level decline were more likely to experience recurrence. By clarifying the significance of HDR-BT in very high-risk PC management, this study informs future research directions focused on mitigating the risk of systemic failure and improving oncological outcomes.

## 1. Introduction

In the most recent update to the National Comprehensive Cancer Network (NCCN) Guidelines, the criteria for the “very high-risk” (VHR) prostate cancer (PC) category were substantially revised by identifying patients with at least two of the following features as a VHR group: clinical stage cT3-cT4, Grade Group 4 or 5, or prostate-specific antigen (PSA) level > 40 ng/mL [1]. This update has significant clinical implications, as the new VHR definition identifies a population with more aggressive tumor biology and worse prognosis than the former definition. Radiotherapy (RT) combined with long-term androgen deprivation therapy (ADT) is generally recommended for patients with regional disease (staged as cN1) or newly defined VHR PC [2,3,4]. However, the potential role of treatment intensification, such as androgen receptor pathway inhibitors (ARPIs) and taxane-based chemotherapy, in combination with definitive RT remains unclear in this population and is the focus of ongoing randomized trials.

High-dose-rate brachytherapy (HDR-BT) offers distinct radiobiological and technical advantages compared with other available RT modalities for unfavorable-risk PC. It enables maximal tumor coverage while sparing adjacent organs at risk by delivering highly conformal, hypofractionated irradiation with steep dose gradients, resulting in high tumor control and acceptable toxicity profiles [5,6,7,8,9]. Several retrospective studies in Japan have demonstrated the efficacy of HDR-BT combined with external beam RT (EBRT) and ADT in patients with high-risk or locally advanced PC [10,11,12,13,14,15]. However, these studies predominantly included heterogeneous high-risk patients and did not specifically evaluate outcomes based on the updated NCCN risk stratification.

Although HDR-BT has been increasingly considered a potent treatment option, robust evidence of its long-term oncological outcomes in the newly defined VHR category or in regional disease is rare. To the best of our knowledge, no previous study has investigated HDR-BT outcomes exclusively among patients classified as VHR according to the recent NCCN Guidelines, although this revised definition encompasses a distinctly more severe patient cohort than earlier classifications. Additionally, studies on HDR-BT outcomes in patients with regional disease are rare, thereby limiting the evidence base for this challenging subgroup.

Therefore, this study aimed to evaluate the long-term clinical outcomes of HDR-BT in patients with VHR and regional PC, as defined by the latest NCCN Guidelines. By focusing on this highly aggressive and newly redefined subset, the findings can help clarify the therapeutic potential and contemporary role of HDR-BT in definitive treatment strategies for one of the highest-risk forms of PC.

## 2. Materials and Methods

### 2.1. Study Design and Patients

A total of 574 consecutive patients with PC who underwent HDR-BT combined with EBRT at Kanazawa University Hospital between January 2006 and December 2022 were enrolled in this study. Of the 574 patients, 215 with VHR or regional PC according to the recent NCCN Guidelines who had a follow-up period of ≥2 years were included in this retrospective analysis. Patients with VHR PC were further stratified into two groups: those meeting two risk factors (VHR2) and those meeting all three risk factors (VHR3). Finally, of the 215 patients, 145 were classified as the VHR2 group, 34 as the VHR3 group, and 36 as the regional group. Clinical staging was categorized according to the tumor-node-metastasis classification [16].

### 2.2. Treatment Protocol

#### 2.2.1. HDR-BT

**Figure 1:**
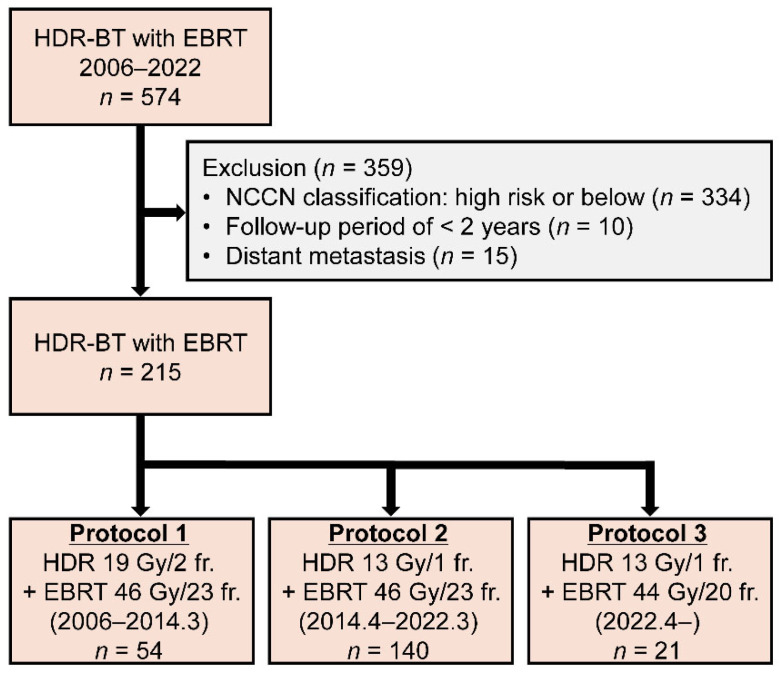
Flow diagram of patient inclusion in the study.

The HDR-BT protocol has been previously documented [17,18]. Patients were excluded from HDR-BT based on several anatomical and technical constraints to ensure treatment safety and quality. Specific exclusion criteria included: (1) prostate volume < 10 cc, where optimal needle spacing and dose distribution were technically unfeasible; (2) severe pubic arch interference predicted by pre-procedural imaging, particularly in large prostates (>50 cc), which obstructed the transperineal needle path; (3) extensive tumor cephalad extension (e.g., near the sigmoid colon) that exceeded the reach of transperineal needles or posed an unacceptable risk of bowel injury; and (4) morbid obesity, where the thickness of perineal subcutaneous tissue precluded stable and sufficient needle penetration into the target volume. HDR-BT was provided to patients with regional lymph node metastasis irrespective of nodal size or number, provided that the disease was confined within the pelvic radiation field. However, it is not suitable for metastases outside the pelvic external irradiation field. All patients received EBRT combined with HDR-BT, with treatment regimens that evolved as shown in Figure 1. From January 2006 to March 2014, HDR-BT was administered at 9.5 Gy in 2 fractions combined with EBRT of 2 Gy in 23 fractions, yielding a biologically effective dose (BED, α/β = 1.5) of 241 Gy (protocol 1). From April 2014 to March 2022, the HDR-BT regimen was modified to a single fraction of 13 Gy while maintaining EBRT at 2 Gy in 23 fractions, resulting in a BED of 233 Gy (protocol 2). Since April 2022, HDR-BT has continued as a single fraction of 13 Gy, but EBRT has been administered at 2.2 Gy in 20 fractions, yielding a BED of 234.2 Gy (protocol 3).

#### 2.2.2. EBRT

EBRT was usually started 1 or 2 weeks after HDR-BT. EBRT was delivered using either intensity-modulated RT or volumetric modulated arc therapy in the prone position. The EBRT procedures followed the established methodology extensively described in our previous work [17,18].

#### 2.2.3. ADT

Neoadjuvant hormonal therapy (HT) was achieved using a combination of bicalutamide (80 mg/day) and a gonadotropin-releasing hormone agonist (11.25 mg leuprorelin or 10.8 mg goserelin). This hormonal intervention was maintained for a 6-month period preceding the HDR-BT procedure. Adjuvant HT was administered for 2 years in patients with ≥2 high-risk factors based on localized high-risk categories according to the NCCN Guidelines (≥cT3 or ≥Grade Group 4 or PSA > 20 ng/mL) or locally advanced PC (≥cT3b).

### 2.3. Toxicity Evaluation

Adverse events associated with the treatment were meticulously recorded in accordance with the Common Terminology Criteria for Adverse Events version 5.0 to facilitate a detailed safety assessment.

### 2.4. Statistical Analyses

Recurrence-free survival (RFS), cancer-specific survival (CSS), and overall survival (OS) were calculated from the initiation of brachytherapy using the Kaplan–Meier method. Differences in RFS across various clinical oncological parameters were assessed using the log-rank and log-rank trend tests. The differences between the patients’ clinicopathological characteristics were compared using Chi–squared test and Kruskal–Wallis test appropriately. Recurrence was defined as the occurrence of any of the following: biochemical recurrence based on the Phoenix criteria (PSA nadir + 2 ng/mL) [19]; radiographic recurrence; or salvage HT initiation. Radiological imaging modalities used to identify the sites of recurrence included conventional computed tomography, magnetic resonance imaging, and bone scintigraphy. The times to RFS and OS were evaluated using univariate and multivariate Cox proportional hazards regression models. Statistical analyses were performed using GraphPad Prism (version 6.07; GraphPad Software Inc., San Diego, CA, USA) and Statistical Package for the Social Sciences (version 29; IBM Corp., Armonk, NY, USA). For all tests, a two-sided *p*-values < 0.05 was considered to be statistically significant.

## 3. Results

### 3.1. Patient Characteristics

A total of 215 patients with VHR and regional PC were included in this retrospective study. Table 1 shows the patients’ clinical and pathological features. The median follow-up duration was 5.6 years (range, 2.0–17.3 years), and the median age was 70 years (range, 50–85 years). The median PSA level at diagnosis was 22.80 ng/mL (range, 0.93–2465.76 ng/mL). PSA levels were ≤20 ng/mL in 91 (42.3%) patients, >20–40 ng/mL in 56 (26.0%) patients, and >40 ng/mL in 68 (31.6%) patients. The median pre-RT PSA level measured within 1 month of HDR-BT was 0.07 ng/mL (range, 0–25.44 ng/mL). PSA levels were ≤0.1 ng/mL in 134 (62.3%) patients and >0.1 ng/mL in 81 (37.7%) patients. Grade Group was ≤3 in 18 (8.4%) patients, 4 in 101 (47.0%) patients, and 5 in 96 (44.7%) patients. Clinical T stage was ≤cT3a in 96 (44.7%) patients, cT3b in 85 (39.5%) patients, and cT4 in 34 (15.8%) patients.

### 3.2. Treatment Outcomes

**Figure 2:**
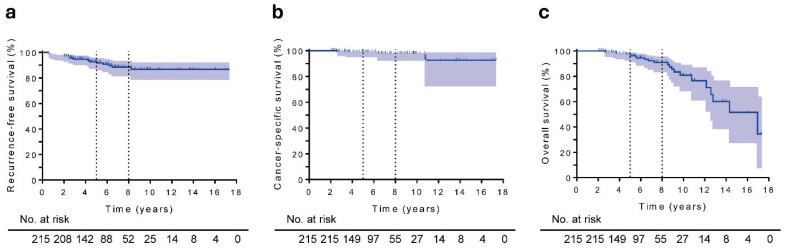
Kaplan–Meier estimates for all patients (The median follow-up period was 5.6 years): (**a**) RFS, (**b**) CSS, and (**c**) OS.

During the observation period, 19 (8.8%) patients had disease recurrence (5 with radiographic recurrence, 11 with biochemical recurrence, and 3 with salvage HT initiation). While the bone was the most common site of radiographic recurrence, no cases of local prostate recurrence were observed. Of the 215 patients, 22 (10.2%) died, including four PC-specific deaths. The 5-year RFS, CSS, and OS rates in the entire cohort were 92.5% (95% confidence interval [CI], 87.6–95.5), 98.9% (95% CI, 95.5–99.7), and 96.2% (95% CI, 91.7–98.3), respectively, at a 5-year follow-up (Figure 2a–c). The 8-year RFS, CSS, and OS rates were 88.5% (95% CI, 81.8–92.8), 97.8% (95% CI, 92.7–99.3), and 90.8% (95% CI, 83.6–95.0), respectively.

**Figure 3:**
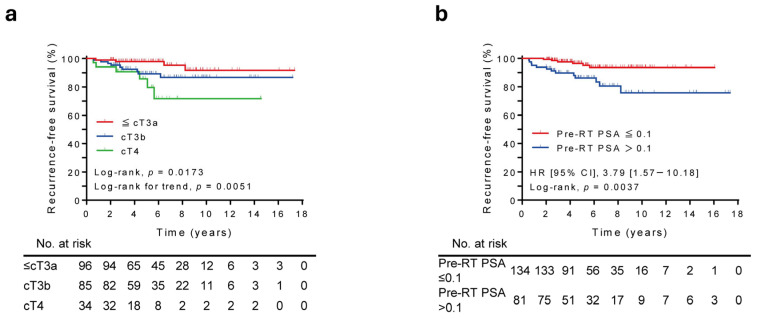
Kaplan–Meier estimates of RFS stratified by (**a**) clinical T stage and (**b**) pre-RT PSA level (The median follow-up period for the entire cohort was 5.3 years).

Table 2 shows the results of the univariate and multivariate Cox proportional hazard regression analyses for the entire cohort. The univariate analysis revealed that elevated pre-RT PSA level, cT4 status, and regional subclassification were significantly associated with RFS. The multivariate analysis revealed that pre-RT PSA level > 0.1 ng/mL (hazard ratio [HR], 3.93; 95% CI, 1.39–11.10; *p* = 0.010) and cT4 status (HR, 4.49; 95% CI, 1.14–17.75; *p* = 0.032) were independent poor predictors for RFS. Age was the only prognostic factor for OS (HR, 2.65; 95% CI, 1.04–6.72; *p* = 0.041). The Kaplan–Meier curves showed significantly inferior RFS in patients with higher clinical T stage (log-rank, *p* = 0.0173; log-rank for trend, *p* = 0.0051) and pre-RT PSA level (log-rank, *p* = 0.0037) (Figure 3a,b). The 5-year RFS according to clinical T stage was 97.8% in patients with ≤cT3a, 89.3% in patients with cT3b, and 85.7% in patients with cT4. Conversely, the 5-year RFS according to pre-RT PSA level was 96.4% in patients with pre-RT PSA ≤ 0.1 and 86.2% in patients with pre-RT PSA > 0.1. According to clinical and oncological parameters, patients with a poor NCCN risk classification exhibited significantly lower RFS rates, as evidenced by Kaplan–Meier survival curves. (log-rank, *p* = 0.0466; log-rank for trend, *p* = 0.0136) (Figure S1). Conversely, age, initial PSA level, Grade Group, and RT protocol showed no significant association with RFS.

### 3.3. Toxicity

Table 3 shows the results of treatment-related toxicity. Genitourinary toxicity was mostly low-grade. Overall, Grade 1 and Grade 2 events occurred in 34.0% and 12.6% of patients, respectively, whereas Grade ≥ 3 events were rare (1.9%) in the entire cohort. Regarding Grade ≥ 3 toxicities, two cases of hematuria were managed with blood transfusion or endoscopic electrocoagulation. The case of prostatitis required intravenous antibiotic therapy, and the case of urethral stricture was treated with internal urethrotomy. All events were successfully resolved following these clinical interventions without long-term sequelae.

Gastrointestinal toxicity was even less common. Grade 1 and Grade 2 events occurred in 12.1% and 2.3% of patients, respectively, and no Grade ≥ 3 gastrointestinal toxicity occurred. The incidence of Grade ≥ 2 events was low, and severe adverse events were exceedingly rare.

## 4. Discussion

This study is the first to evaluate the outcomes of HDR-BT in patients with VHR or regional disease as defined by the updated NCCN criteria. Definitive RT incorporating HDR-BT achieved durable disease control in this single-institution cohort of 215 men with VHR or regional PC. The 5-year RFS, CSS, and OS rates were 92.5%, 98.9%, and 96.2%, respectively. These rates remained favorable at 8 years (88.5%, 97.8%, and 90.8%, respectively), supporting the premise that the HDR-BT-based approach can deliver oncologic outcomes commensurate with the biologic aggressiveness of this subgroup. These results are consistent with those of previous studies, which showed that HDR-BT improves local tumor control and biochemical outcomes in patients with high-risk and VHR disease [10,11,12,20,21,22]. Importantly, this study focused exclusively on patients classified as having VHR or regional disease according to the updated NCCN definitions.

Previous studies have shown the potential advantages of HDR-BT as a boost in combination with EBRT and ADT. Kasahara et al. reported a 5-year biochemical failure-free survival rate of 88.7% in VHR patients [10]. Similarly, Mori et al. reported a 5-year biochemical RFS rate of 70.0% after HDR-BT combined with EBRT and ADT [12]. Additionally, long-term outcomes over a 24-year follow-up period showed sustained biochemical control (68%) despite unfavorable disease characteristics [23]. A notable strength of HDR-BT is its superior dosimetric profile, enabling highly conformal and homogeneous intraprostatic dose escalation compared with EBRT alone. Such precision may improve local control, particularly in tumors with large intraprostatic burden or adverse features. Although the findings of previous studies are based on the definitions in the previous NCCN Guidelines, our results compare favorably despite restricting the cohort to patients with VHR and regional PC based on the definitions in the latest NCCN Guidelines, indicating that HDR-BT can provide effective disease control in this population. However, it is essential to recognize that HDR-BT is not a uniform procedure, but a complex intervention characterized by significant technical and multidisciplinary demands. The clinical success and reproducibility of this approach are inherently linked to factors such as implant technique, the number of catheters used, the precision of imaging guidance, and anesthesia requirements. Furthermore, the workflow and resource allocation are heavily influenced by the expertise of the multidisciplinary team, including urologists, radiation oncologists, and radiological technologists. While our institution maintained consistent protocol and we saw increasing institutional experience and organizational refinement in our multidisciplinary teams, the inherent heterogeneity in procedural complexity across different centers may impact the scalability of these results. Acknowledging HDR-BT as a sophisticated, operator-dependent modality is crucial for placing our findings within a broader real-world clinical context [24].

This study showed a low overall recurrence rate (8.8%), with bone being the predominant site of radiographic recurrence. These patterns of failure indicate that, when robust local control is achieved, the remaining events are largely systemic. This aligns with the rationale for combining high-quality local therapy with long-term ADT in VHR and regional disease. Notably, only four PC-specific deaths occurred during follow-up, whereas 22 total deaths occurred, indicating effective cancer control and highlighting the impact of competing, age-related mortality in this population.

While our results underscore the efficacy of HDR-BT combined with EBRT, it is essential to contextualize these findings relative to other treatment modalities such as radical prostatectomy (RP) and stereotactic body RT (SBRT). For high-risk and VHR PC, HDR-BT boost has been reported to offer superior metastasis-free survival or biochemical RFS compared to EBRT alone or RP in certain cohorts [25,26]. Furthermore, in comparison to SBRT boost, HDR-BT allows for superior dose escalation and conformality by direct source placement within the prostate. This provides a distinct advantage in managing T3b-T4 or node-positive disease, where maximizing the dose to the extraprostatic extension while sparing adjacent critical organs is paramount [27]. Thus, our long-term outcomes suggest that HDR-BT remains a robust and highly competitive option within the multidisciplinary management of VHR PC.

Prognostic analyses highlight the centrality of baseline disease burden and local extent. In multivariate modeling, elevated pre-RT PSA and cT4 were independent predictors of inferior RFS (HR 3.93 and 4.49, respectively), whereas age was the only predictor of OS. These findings have several implications. First, pre-RT PSA, reflecting initial tumor burden and the degree of response to neoadjuvant ADT, emerges as a pragmatic surrogate of residual risk at the time of definitive RT. Second, despite dose-intense local therapy, cT4 disease retains adverse prognostic significance, highlighting the persistent challenge of achieving adequate pelvic control and underscoring the need for integration of systemic intensification strategies in this subgroup. Third, although regional classification was associated with RFS in the univariate analysis, it did not remain an independent predictor after adjustment, indicating collinearity with other indices of burden and extent. Finally, the finding that age was the only independent predictor of OS may reflect the very low number of PC-specific deaths in this cohort, with most deaths arising from noncancer causes. Consequently, competing mortality diminishes the influence of oncologic factors on OS, leaving age as the dominant determinant.

These results are consistent with and extend our recent work [28], which showed that baseline PSA and T stage consistently emerged as dominant prognostic determinants across all PCs. This study builds on these findings by focusing specifically on patients with NCCN-defined VHR and regional disease treated with HDR-BT-based therapy, thereby confirming the robustness of these prognostic markers in an intensively treated cohort. These findings underscore the clinical utility of PSA and T stage as reproducible risk indicators and highlight the value of integrating them into risk-adapted strategies that may inform local and systemic treatment intensification.

The study strengths lie in its relatively large VHR/regional cohort, consistent application of modern risk definitions, and long follow-up (median, 5.6 years; maximum, >17 years). Despite these strengths, this study has some limitations that warrant consideration. The retrospective, single-institution design of this study introduces potential selection bias and residual confounding. Additionally, the study period spans more than a decade, during which HDR-BT and EBRT techniques evolved, resulting in some variations in dose and fractionation. Although this heterogeneity reflects real-world practice and broadens generalizability, it complicates attributing outcomes to any single regimen. However, all approaches delivered biologically comparable doses for high-risk disease, and institutional protocols ensured consistent target coverage and quality assurance throughout the study period, thereby mitigating the impact of treatment heterogeneity on the primary oncologic endpoints. Pathological grading and clinical staging were performed in routine practice. However, our long-term results may be affected by ‘stage migration’ arising from evolving diagnostic standards. The transition to multiparametric magnetic resonance imaging and the lack of uniform prostate-specific membrane antigen positron emission tomography (PSMA-PET) imaging during the study period likely influenced staging precision compared to contemporary practice, particularly for regional disease. Furthermore, the relatively small number of clinical events within our cohort may have limited the statistical power of the Cox proportional hazards models. This low event rate, while reflective of the favorable oncological outcomes associated with our treatment protocol, necessitates caution when interpreting the precision of the identified prognostic factors. Additionally, as this cohort consists exclusively of Japanese patients, the generalizability of our findings to other ethnic populations remains to be validated. Finally, although cancer control outcomes are robust, quality-of-life endpoints were not analyzed in this study and should be addressed in future studies to fully define the therapeutic index of this approach.

Future research should incorporate insights from ongoing randomized trials evaluating treatment intensification for high-risk and VHR PC. Clinical trials such as DASL-HiCaP (NCT04136353) and ATLAS (NCT02531516) are assessing the benefit of adding ARPIs to RT and ADT to improve systemic control beyond that achieved with conventional HT [29,30]. Similarly, PEACE-2 (NCT01952223) and GETUG-P17/ALADDIN (NCT05116475) are evaluating the addition of taxane-based chemotherapy or darolutamide to multimodal systemic intensification strategies [31,32]. For patients with extremely high-risk features, such as cT4 disease or suboptimal pre-RT PSA response, these intensified systemic strategies should be tailored to complement the high-dose intensity of HDR-BT. The synergistic effect of potent local control and early systemic escalation could be crucial in mitigating the bone-predominant systemic failures observed in our study, thereby addressing both local progression and micrometastatic disease in these challenging subgroups. Future prospective evaluations are warranted to refine the therapeutic index of HDR-BT-based strategies. These studies should encompass modern imaging modalities like PSMA-PET, uniform HDR-BT/EBRT delivery, and patient-reported outcomes, particularly for the VHR and regional disease subsets.

## 5. Conclusions

Definitive RT incorporating HDR-BT shows promising long-term control in patients with VHR and regional PC as defined by the latest NCCN criteria. Elevated pre-RT PSA and cT4 disease can identify patients at an increased risk of recurrence and may inform individualized decisions regarding systemic therapy and surveillance intensity. These findings support the role of HDR-BT as a cornerstone of multimodality treatment for one of the most aggressive localized and locally advanced PC phenotypes.

## Figures and Tables

**Table 1:** Patients characteristics.

	All Patients	VHR2	VHR3	Regional	*p*-Value
Number of patients	215	145	34	36	
Follow-up (years)					0.180
Median (range)	5.6 (2.0–17.3)	5.6 (2.0–17.3)	5.8 (2.0–17.0)	5.0 (2.1–14.0)	
Age (year), *n* (%)					0.232
Median (range)	70 (50–85)	70 (54–85)	71 (59–85)	71 (50–80)	
≤70	115 (53.5)	82 (56.6)	15 (44.1)	18 (50)	
>70	100 (46.5)	63 (43.4)	19 (55.9)	18 (50)	
PSA at diagnosis (ng/mL), *n* (%)					<0.001
Median (range)	22.80 (0.93–2465.76)	16.00 (0.93–458.62)	61.46 (40.16–2465.76)	31.77 (2.04–557.64)	
≤20	91 (42.3)	81 (55.9)	0	10 (27.8)	
20<, ≤40	56 (26)	46 (31.7)	0	10 (27.8)	
40<	68 (31.6)	18 (12.4)	34 (100)	16 (44.4)	
Grade Group, *n* (%)					0.171
≤3	18 (8.4)	12 (8.3)	0	6 (16.7)	
4	101 (47)	69 (47.6)	17 (50)	15 (41.7)	
5	96 (44.7)	64 (44.1)	17 (50)	15 (41.7)	
Clinical T stage, *n* (%)					<0.001
≤cT3a	96 (44.7)	79 (54.5)	11 (32.4)	6 (16.7)	
cT3b	85 (39.5)	52 (35.9)	15 (44.1)	18 (50)	
cT4	34 (15.8)	14 (9.7)	8 (23.5)	12 (33.3)	
Pre-RT PSA (ng/mL), *n* (%)					0.007
Median (range)	0.07 (0–25.44)	0.05 (0–10.07)	0.10 (0–25.44)	0.08 (0–6.44)	
≤0.1	134 (62.3)	97 (66.9)	17 (50)	20 (55.6)	
>0.1	81 (37.7)	48 (33.1)	17 (50)	16 (44.4)	
Adjuvant HT, *n* (%)					0.004
Yes	194 (90.2)	124 (85.5)	34 (100)	36 (100)	
RT protocol, *n* (%)					0.027
Protocol 1	54 (25.1)	42 (29)	9 (26.5)	3 (8.3)	
Protocol 2	140 (65.1)	87 (60)	25 (73.5)	28 (77.8)	
Protocol 3	21 (9.8)	16 (11)	0	5 (13.9)	

EBRT, external beam radiation therapy; HDR-BT, high-dose-rate brachytherapy; HT, hormonal therapy; PSA, prostate-specific antigen; RT, radiotherapy; VHR, very high-risk.

**Table 2:** Prognostic factors for RFS and OS.

Covariant		RFS				OS			
		Univariate		Multivariate		Univariate		Multivariate	
		HR (95% CI)	*p*-Value	HR (95% CI)	*p*-Value	HR (95% CI)	*p*-Value	HR (95% CI)	*p*-Value
Age (year)	≤70	Reference		Reference		Reference		Reference	
	>70	1.35 (0.55–3.33)	0.513	1.55 (0.58–4.12)	0.383	2.65 (1.10–6.38)	0.030	2.65 (1.04–6.72)	0.041
PSA at diagnosis (ng/mL)	≤20	Reference		Reference		Reference		Reference	
	20<, ≤40	1.42 (0.46–4.42)	0.544	0.89 (0.26–3.05)	0.855	1.88 (0.61–5.81)	0.273	1.85 (0.58–5.84)	0.296
	40<	1.44 (0.48–4.30)	0.510	0.31 (0.06–1.61)	0.161	1.85 (0.62–5.56)	0.272	1.38 (0.34–5.66)	0.657
Pre-RT PSA (ng/mL)	≤0.1	Reference		Reference		Reference		Reference	
	>0.1	3.79 (1.44–9.98)	0.007	3.93 (1.39–11.10)	0.010	1.21 (0.50–2.91)	0.674	1.16 (0.44–3.05)	0.765
Clinical T stage	≤cT3a	Reference		Reference		Reference		Reference	
	cT3b	2.63 (0.81–8.54)	0.108	2.83 (0.83–9.66)	0.097	0.97 (0.39–2.41)	0.956	1.16 (0.45–2.95)	0.758
	cT4	5.53 (1.55–19.78)	0.008	4.49 (1.14–17.75)	0.032	1.15 (0.31–4.25)	0.839	1.09 (0.24–4.91)	0.910
Grade Group	≤4	Reference		Reference		Reference		Reference	
	5	1.06 (0.43–2.62)	0.893	0.78 (0.31–2.01)	0.610	0.72 (0.30–1.72)	0.456	0.71 (0.27–1.88)	0.495
NCCN risk group	VHR2	Reference		Reference		Reference		Reference	
	VHR3	1.99 (0.61–6.48)	0.253	2.94 (0.52–16.56)	0.221	1.07 (0.35–3.22)	0.906	0.81 (0.19–3.49)	0.772
	Regional	3.44 (1.21–9.73)	0.020	2.80 (0.89–8.86)	0.080	0.86 (0.19–3.77)	0.838	0.88 (0.18–4.31)	0.878

CI, confidence interval; HR, hazard ratio; NCCN, National Comprehensive Cancer Network; PSA, prostate-specific antigen; RT, radiotherapy; VHR, very high-risk.

**Table 3:** Toxicity.

	Grade 1, *n* (%)	Grade 2, *n* (%)	Grade 3, *n* (%)	Total, *n* (%)
Genitourinary				
VHR2	47 (32.4)	21(14.5)	4 (2.8)	72 (49.7)
VHR3	10 (29.4)	3 (8.8)	0	13 (38.2)
Regional	16 (44.4)	3 (8.3)	0	19 (52.8)
Total	73 (34.0)	27 (12.6)	4 (1.9)	104 (48.4)
Gastrointestinal				
VHR2	17 (11.7)	4 (2.8)	0	21 (14.5)
VHR3	3 (8.8)	0	0	3 (8.8)
Regional	6 (16.7)	1 (2.8)	0	7 (19.4)
Total	26 (12.1)	5 (2.3)	0	31 (14.4)

VHR, very high-risk.

## Data Availability

Data are contained within the article or its Supplementary Material.

## Supplementary Materials

The following supporting information can be downloaded at: https://www.mdpi.com/article/10.3390/cancers18040595/s1, Figure S1, Recurrence-free survival after HDR-BT stratified by (a) age, (b) initial PSA, (c) Grade Group (GG), (d) NCCN risk group, and (e) RT protocol.

## Abbreviations

The following abbreviations are used in this manuscript:

ADT	Androgen deprivation therapy
CSS	Cancer-specific survival
EBRT	External beam radiotherapy
HDR-BT	High-dose-rate brachytherapy
HT	Hormonal therapy
NCCN	National Comprehensive Cancer Network
OS	Overall survival
PC	Prostate cancer
PSA	Prostate-specific antigen
PSMA-PET	Prostate-specific membrane antigen positron emission tomography
RFS	Recurrence-free survival
RP	Radical prostatectomy
RT	Radiotherapy
SBRT	Stereotactic body radiotherapy
VHR	Very high-risk
