# Supplementary material for: High-Dose-Rate Brachytherapy Combined with External Beam Radiotherapy for Newly Defined Very High-Risk and Regional Prostate Cancer: A 17-Year Single-Institution Experience
Source: Cancers (Basel). 2026 Feb 11;18(4):595. doi: 10.3390/cancers18040595 (PMC12938564; doi:10.3390/cancers18040595)
Supplement: Supplementary file 1 [file cancers-18-00595-s001.zip › cancers-4117007-supplementary.pptx]

## Slide 1
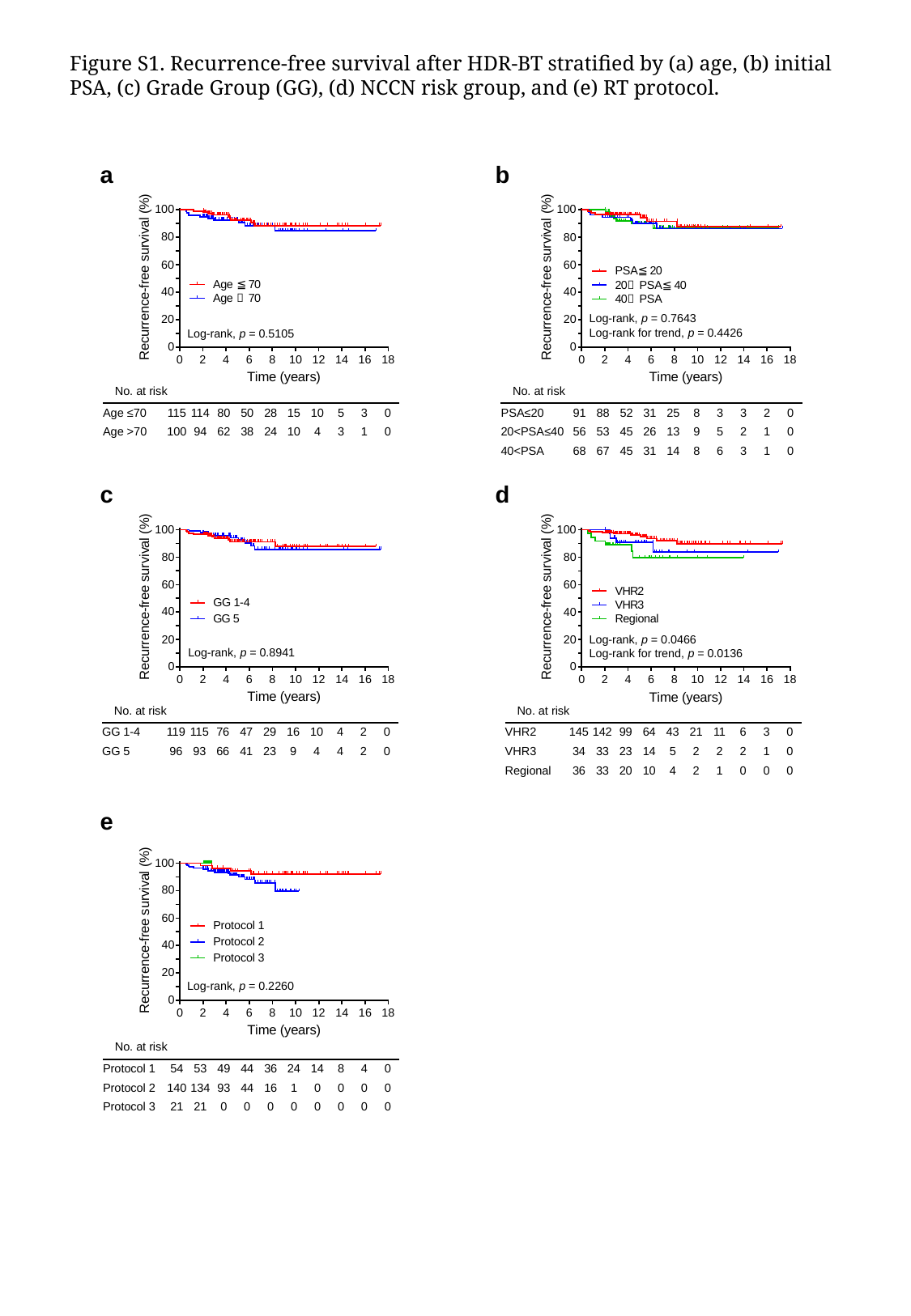

Figure S1. Recurrence-free survival after HDR-BT stratified by (a) age, (b) initial PSA, (c) Grade Group (GG), (d) NCCN risk group, and (e) RT protocol.
a
b
No. at risk
No. at risk
| Age ≤70 | 115 | 114 | 80 | 50 | 28 | 15 | 10 | 5 | 3 | 0 |
| --- | --- | --- | --- | --- | --- | --- | --- | --- | --- | --- |
| Age >70 | 100 | 94 | 62 | 38 | 24 | 10 | 4 | 3 | 1 | 0 |
| PSA≤20 | 91 | 88 | 52 | 31 | 25 | 8 | 3 | 3 | 2 | 0 |
| --- | --- | --- | --- | --- | --- | --- | --- | --- | --- | --- |
| 20<PSA≤40 | 56 | 53 | 45 | 26 | 13 | 9 | 5 | 2 | 1 | 0 |
| 40<PSA | 68 | 67 | 45 | 31 | 14 | 8 | 6 | 3 | 1 | 0 |
c
d
No. at risk
No. at risk
| GG 1-4 | 119 | 115 | 76 | 47 | 29 | 16 | 10 | 4 | 2 | 0 |
| --- | --- | --- | --- | --- | --- | --- | --- | --- | --- | --- |
| GG 5 | 96 | 93 | 66 | 41 | 23 | 9 | 4 | 4 | 2 | 0 |
| VHR2 | 145 | 142 | 99 | 64 | 43 | 21 | 11 | 6 | 3 | 0 |
| --- | --- | --- | --- | --- | --- | --- | --- | --- | --- | --- |
| VHR3 | 34 | 33 | 23 | 14 | 5 | 2 | 2 | 2 | 1 | 0 |
| Regional | 36 | 33 | 20 | 10 | 4 | 2 | 1 | 0 | 0 | 0 |
e
No. at risk
| Protocol 1 | 54 | 53 | 49 | 44 | 36 | 24 | 14 | 8 | 4 | 0 |
| --- | --- | --- | --- | --- | --- | --- | --- | --- | --- | --- |
| Protocol 2 | 140 | 134 | 93 | 44 | 16 | 1 | 0 | 0 | 0 | 0 |
| Protocol 3 | 21 | 21 | 0 | 0 | 0 | 0 | 0 | 0 | 0 | 0 |
